# Environmental sampling for SARS-CoV-2 in long term care facilities: lessons from a pilot study

**DOI:** 10.12688/wellcomeopenres.17047.2

**Published:** 2023-06-28

**Authors:** Rachel Kwiatkowska, Nicola Yaxley, Ginny Moore, Allan Bennett, Matthew Donati, Nicola Love, Roberto Vivancos, Matthew Hickman, Derren R Ready

**Affiliations:** 1 Population Health Sciences, Bristol Medical School, University of Bristol, Bristol, UK; 2 NIHR Health Protection Research Unit in Behavioural Science and Evaluation, University of Bristol, Bristol, UK; 3 Field Service, Health Protection Operations, UKHSA, Bristol, UK; 4 Biosafety, Air and Water Microbiology Group, UKHSA, Porton Down, Salisbury, UK; 5 Severn Infection Sciences, North Bristol NHS Trust, Bristol, UK; 6 UKHSA Specialised Microbiology and Laboratories,, South West Regional Laboratory, Bristol, UK; 7 Field Service, Health Protection Operations, UKHSA, Newcastle, UK; 8 NIHR Health Protection Research Unit in Gastrointestinal Infections, University of Liverpool, Liverpool, UK; 9 Field Service, Health Protection Operations, UKHSA, Liverpool, UK; 10 Eastman Dental Institute, University College London, London, UK

**Keywords:** infection control; infectious disease transmission; environmental exposure; fomites; disease outbreaks; long-term care; epidemiologic methods

## Abstract

**Background:** The SARS-CoV-2 pandemic has highlighted the risk of infection in long-term care facilities (LTCF) and the vulnerability of residents to severe outcomes. Environmental surveillance may help detect pathogens early and inform Infection Prevention and Control (IPC) measures in these settings.

**Methods:** Upon notification of SARS-CoV-2 outbreaks, LTCF within a local authority in South West England were approached to take part in this pilot study. Investigators visited to swab common touch-points and elevated ‘non-touch’ surfaces (>1.5m above ground level) and samples were analysed for presence of SARS-CoV-2 genetic material (RNA). Data were collected regarding LTCF infrastructure, staff behaviours, clinical and epidemiological risk factors for infection (staff and residents), and IPC measures.

Criteria for success were: recruitment of three LTCF; detection of SARS-COV-2 RNA; variation in proportion of SARS-CoV-2 positive surfaces by sampling zone; and collection of clinical and epidemiological data for context.

**Results:** Three LTCFs were recruited, ranging in size and resident demographics. Outbreaks lasted 63, 50 and 30 days with resident attack rates of 53%, 40% and 8%, respectively. The proportion of sample sites on which SARS-CoV-2 was detected was highest in rooms occupied by infected residents and varied elsewhere in the LTCF, with low levels in a facility implementing enhanced IPC measures. The heterogeneity of settings and difficulty obtaining data made it unfeasible to assess association between environmental contamination and infection. A greater proportion of elevated surfaces tested positive for SARS-CoV-2 RNA than common touch-points.

**Conclusions:** SARS-CoV-2 RNA can be detected in a variety of LTCF outbreak settings, both on common-touch items and in elevated sites out of reach. This suggests that further work is justified, to assess feasibility and utility of environmental sampling for infection surveillance in LTCF.

## Introduction

Long term care facilities (LTCF) are inadvertently ideal environments for the spread of pathogens. ( Strausbaugh *et al*., 2003) Residents are often susceptible to infection or colonisation, and in frequent and close contact with staff who have links to the wider community. Outbreaks of infectious diseases are common in these settings ( Inns *et al*., 2017; Inns *et al*., 2019) and the coronavirus 2019 (COVID-19) pandemic, caused by SARS-CoV-2 coronavirus, has highlighted the vulnerability of people in LTCF to infectious disease threats: almost 30,000 excess deaths are thought t have occurred among LTCF residents in England over the first 23 weeks of the epidemic. ( Morciano *et al*., 2021)

If detected early enough transmission of infections within the LTCF can be curbed, ( Inns *et al*., 2018) however SARS-CoV-2 infections are often asymptomatic or paucisymptomatic leading to large outbreaks. Regular clinical testing of residents and staff helps identify cases early but is resource-intensive and unpleasant for frail individuals, so non-invasive surveillance strategies may be more sustainable Respiratory pathogens have been identified from surfaces in various healthcare and non-healthcare settings: typically on objects that have been contaminated by touch, but also surfaces such as window ledges, ventilation grilles and ventilation filters which airborne pathogens will settle on. ( Goyal *et al.*, 2011; Moore *et al.*, 2021; Santarpia *et al.*, 2020). The authors also identified SARS-CoV-2 RNA on the top of staff lockers in a hospital setting and a university workshop in the early phases of the pandemic (unpublished data). Swabbing common-touch and non-touch surfaces within LTCF could signal the presence of infection as well as providing insights into how the virus is transmitted, which can inform infection prevention and control (IPC) measures. According to a World Health Organization scientific brief on transmission of SARS-CoV-2 (2020), direct (droplet) transmission and indirect spread via fomites (contaminated surfaces) and long-distance aerosols are thought to occur; however there is no conclusive evidence for indirect transmission in LTCF. ( Ben-Shmuel *et al*., 2020; Greenhalgh *et al*., 2021; Ong *et al*., 2020; Ye *et al*., 2020)

COVID-19: Detecting Indirect Transmission in Facilities for Enhanced Care sTudy (COVID-19: DISinFECT) aims to investigate the role of indirect transmission of SARS-CoV-2 in LTCF and assess whether environmental surveillance could inform IPC measures in these settings. The idea was to conduct environmental sampling (surface swabs, air sampling and wastewater) during outbreaks of SARS-CoV-2 infection in LTCFs and assess environmental dispersal of SARS-CoV-2 RNA in the context of a) infections identified through enhanced clinical testing of residents and staff; b) clinical and epidemiological risk factors for infection; and c) different infection prevention and control measures. We present findings from a pilot conducted between 14 ^th^ January and 28 ^th^ March 2021, during the second epidemic wave in South West England.

## Methods

The full DISinFECT protocol can be accessed online. ( Kwiatkowska & Ready, 2021) LTCFs were eligible for inclusion if they provided residential care for older adults (>65 years), were within the boundaries of a selected local authority in the UKHSA (UK Health Security Agency, formerly Public Health England South West region, and experienced a COVID-19 outbreak: defined as two or more laboratory-confirmed cases among staff and/or residents within a 14-day period.

### Recruitment

On notification of an outbreak, investigators contacted the LTCF manager with information about DISinFECT and offered environmental sampling as part of outbreak management. If managers expressed an interest, they were asked to complete a written consent form permitting the study team to conduct telephone interviews, collect information from LTCF records, sample the LTCF environment and approach residents and staff for involvement. Prior to the sampling visit, residents and staff were provided with written and pictorial leaflets describing the purpose of the investigations, sampling procedures and how their information would be processed. Each of the residents selected for sampling was consulted to make sure they understood this information and were happy to provide samples. Sampling was not carried out if the individual lacked mental capacity to complete a consent form. Staff were asked for consent to participate prior to accessing the electronic questionnaire.

### Sampling

***Approach***. Settings varied in size and layout but sampling was done systematically, with a focus on a) common touch points (for example: door handles, light switches, television remote controls) and b) elevated surfaces above 1.5m in height, which were unlikely to be touched or regularly cleaned, onto which airborne virus might settle (for example: door sills, tops of wall-mounted cabinets).

Within each LTCF, sampling sites were categorised in to three ‘zones’: 1) rooms occupied by residents isolating with active SARS-CoV-2 infection, or equipment used by them, 2) areas/equipment used by both staff and residents such as lounges and dining areas, shared kitchen equipment, and 3) staff-only areas/equipment such as offices, recreation areas, and key cabinets.

***Materials*.** The majority of surfaces were sampled using Sterilin flocked swabs, supplied in tubes containing 2mL of universal transport medium which was used to wet the swab prior to sampling. Larger areas (e.g., benches/ tabletops) were sampled using pre-moistened TSC Enviroscreen sterile cellulose sponges measuring 50cm ^2^, supplied in stomacher pouches containing 10mL neutralising buffer. Wetted swabs were also used to sample from the fingertips of residents in isolation rooms. All samples were transported to a public health laboratory specialising in aerobiology, biocontainment and biosafety measures.

The full DISinFECT protocol includes sampling stool and saliva from resident cases and air and wastewater sampling from the facility. For logistical reasons, stool and saliva could not be taken during this pilot. Likewise, no air sampling was conducted, and wastewater was collected from only one facility; results will be reported separately.

### Laboratory analysis

***Extraction*.** From each swab/ sponge container, we took a 140μL aliquot of the transport medium/ neutralising buffer. Viral RNA was extracted using a QIAamp Viral RNA Mini Kit (Qiagen Ltd, Manchester, UK), according to the manufacturer’s instructions. From 60μL eluate, 5μL extracts were used for analysis. Each set of around 15 extractions included at least one negative (ethanol) extraction control.

***Analysis*.** Each sample was analysed in duplicate using a Reverse Transcription Polymerase Chain Reaction (RT-PCR) assay targeting both the N gene and the ORF1ab gene of SARS-CoV-2 (Viasure, CerTest Biotec, Zaragoza). We used an Applied Biosystems QuantStudio 5 thermocycler.

RT-PCR results were reported in cycle threshold (Ct) numbers. A sample was classified as ‘positive’ for SARS-CoV-2 if amplification of one or both targets was detected in both duplicates; ‘suspect’ if detected in only one duplicate and ‘negative’ if no amplification was detected for either gene target. A Ct value of 39 was used as the cut-off point, as in similar sampling studies in hospitals and care homes. ( Dumont-Leblond *et al.*, 2021; Moore *et al.*, 2021; Nelson *et al.*, 2021).

RT-PCR was repeated (in duplicate) for ‘suspect’ samples. If repeat RT-PCR detected amplification of a gene target in both duplicates, then the ‘suspect’ sample was reclassified as ‘positive’.

A PCR reaction efficiency internal control was included in the qPCR mastermix, to assess for inhibition. If duplicate Ct values of the internal control had a standard deviation (SD) of >0.5, qPCR was repeated.

Quantification was carried out for swab samples; methods and results are available with underlying data (see below). This paper will focus only on detection or absence of SARS-CoV-2 RNA.

### Whole Genome Sequencing

Whole genome sequencing (WGS) was conducted on a subset of clinical isolates from nose/ throat swabs, to identify SARS-CoV-2 variants and mutations of interest or concern.

### Epidemiological data collection

In addition to sampling, the study team recorded details of LTCF layout, cleaning and IPC measures, and staff and resident behaviours. Clinical test results (nasopharyngeal swabs analysed with PCR) were obtained from the regional public health laboratory and LTCF managers provided additional information about clinical case notifications, resident risk factors for infection and IPC measures. Finally, staff members were sent an electronic questionnaire asking questions about exposures and risk factors for infection.

Criteria for success in the pilot are presented in Box 1.

**Box 1. DISinFECT pilot criteria for success**

a) Recruitment of three LTCF and consent to participate from at least one resident per facility;

b) Detection of SARS-CoV-2 RNA from surface sampling;

c) Variation by sampling zone in the proportion of sample sites testing positive for SARS-CoV-2 RNA;

d) Potential to assess transmission risk from environmental contamination, in the context of individual risk factors for infection.

### Ethical considerations

These investigations were carried out as part of a public health response to the SARS-CoV-2 pandemic. Ethical approval was granted on 14 ^th^ January 2021 by the Public Health England Research Ethics and Governance Group (PHE REGG: RD 415).

## Results

Four LTCFs were approached on notification of an outbreak: one declined to participate as they did not have the capacity to support recruitment and sampling. Three LTCFs were sampled between 2 ^nd^ February and 10 ^th^ March 2021 and a total of 84 environmental swabs were taken (56 from common touch points, 28 from elevated sites). One LTCF had two sampling visits, 14 days apart. For simplicity, we have labelled the L A, B and C in order of sampling dates. Table 1 contains the full list of sampling sites.

**Table 1.** Sampling sites and SARS-CoV-2 RNA positivity by facility.

Facility A: sampling sites	*	Ct (N) †	SARS-CoV-2	Facility B: sampling sites	*	Ct (N) †	2nd visit	SARS-CoV-2	2nd visit	Facility C: sampling sites	*	Ct (N) †	SARS-CoV-2
*Female staff changing room*				*Staff toilet*						*Staff office*			
1. Top of lockers (next to door)	E	ND	Negative	1. Air vent/ extractor	E	ND	ND	Negative	Negative	1. Top of key cupboard	E	ND	Negative
2. Toilet door: inside handle and lock	T	ND	Negative	2. Soap dispenser lever	T	ND	ND	Negative	Negative	2. Computer mouse	T	ND	Negative
3. Air vent/ extractor (toilet)	E	ND	Negative	3. Door ledge (outer)	E	36.80, 36.15	ND	Positive	Negative	3. Top of message board	E	ND	Negative
4. Bench top	T	ND	Negative	*Manager's office*						4. Telephone receiver	T	ND	Negative
5. Soap dispenser lever	T	39.06	Negative	4. Computer mouse	T	ND	ND	Negative	Negative	*Shared toilet (staff/ residents)*			
*Dining room (staff)*				*Laundry room*						5. Air vent/ extractor	E	ND	Negative
6. Top of TV	E	ND	Negative	5. Air vent/ extractor	E	37.42, 37.44	ND	Positive	Negative	6. Soap dispenser lever	T	ND	Negative
7. Top of trolley (for lunch trays)	E	ND	Negative	6. Glove box (size small)	T	ND	N/A	Negative	N/A	*Reception area*			
8. Radio dials	T	ND	Negative	*Shared toilet (staff/ residents)*						7. Front door keypad (inside)	T	ND	Negative
*Sluice room*				7. Toilet booster handles	T	ND	ND	Negative	Negative	8. Top of kitchen door frame (outside)	E	ND	Negative
9. Code pad (door exterior)	T	ND	Negative	8. Air vent/ extractor	E	35.97, 36.46	ND	Positive	Negative	*Staff break area*			
*Breakout room (staff)*				9. Toilet booster underside	T	ND	ND	Negative	Negative	9. Air vent/ extractor	E	ND	Negative
10. Chair arms	T	ND	Negative	*Dining area (residents & staff)*						10. Fire exit sign	E	ND	Negative
11. Air vent/ extractor	E	ND	Negative	10. Tablecloth	T	36.99	ND	Suspect	Negative	11. Door handle (exit to reception)	T	ND	Negative
12. Light switch	T	ND	Negative	11. Tea trolley handles	T	ND	ND	Negative	Negative	12. Bannister	T	ND	Negative
*Corridor*				12. Armchair handles	T	ND	ND	Negative	Negative	*Lounge/ shared kitchen area*			
13. UV cabinet handle	T	ND	Negative	13. Top of picture frame	E	37.05	ND	Suspect	Negative	13. Top of kitchen hatch	E	ND	Negative
*Photocopier room*				14. TV remote control	T	37.45, 37.62	ND	Positive	Negative	14. Chair arms	T	ND	Negative
14. Photocopier digital pad	T	ND	Negative	*Bedroom/ bathroom*						15. Kettle handle & switch	T	ND	Negative
*Bedroom/ bathroom #1*				15. Top of wardrobe	E	36.67, 33.93	35.00, 35.50	Positive	Positive	16. Microwave handle & dial	T	ND	Negative
15. Bedrails (unoccupied)	T	37.43	Suspect	16. Fingertips (L hand)	-	ND	ND	Negative	Negative	17. Air vent/ extractor	E	ND	Negative
16. Fingertips (R hand)	-	ND	Negative	17. Fingertips (R hand)	-	ND			Negative	*Corridor*			
17. Hoist handle	T	ND	Negative	18. TV remote	T	31.79, 31.71	ND	Positive	Negative	18. Glove box – size small	T	ND	Negative
18. Chair seat (occupied)	T	ND	Negative	19. Tabletop (swab)	T	ND	ND	Negative	Negative	19. Lip of PPE cabinet	T	ND	Negative
19. Air vent/ extractor (bedroom)	E	ND	Negative	20. Tabletop (sponge)	T	37.24, 36.17			Positive	20. Air vent/ extractor	E	ND	Negative
20. Catheter stand (bathroom)	T	ND	Negative	21. Chair arms	T	34.90, 34.93	ND	Positive	Negative	21. Hand rail	T	ND	Negative
*Bedroom/ bathroom #2*				22. Walking frame handles	T	34.69, 35.25	ND	Positive	Negative	*Bedroom/ bathroom*			
21. Bed remote control (occupied)	T	37.06, 38.13	Positive	23. Bed head	T	37.58	37.37	Suspect	Suspect	22. Fingertips (R hand)	-	ND	Negative
22. Air vent/ extractor (bedroom)	E	ND	Negative	24. Wardrobe handle	T	37.50	ND	Suspect	Negative	23. Fingertips (L hand)	-	ND	Negative
23. TV control (in use)	T	34.99, 35.48	Positive	25. Bathroom door handle (outer)	T	37.40		Suspect		24. Table top	T	ND	Negative
24. Top of TV	E	35.96, 35.38	Positive	26. Bathroom door handle (inner)	T	37.02		Suspect		25. Chair arms	T	ND	Negative
25. Top of clock	E	ND	Negative	27. Air vent/ extractor (bathroom)	E	34.64, 34.65	36.60	Positive	Suspect	26. Bed remote control	T	ND	Negative
26. Commode seat (bathroom)	T	37.93, 37.63	Positive	28. Toilet seat booster	T	ND	ND	Negative	Negative	27. Bed head	T	ND	Negative
27. Fingertips (both hands)	-	ND	Negative	*Corridor*						28. Walking frame handles	T	ND	Negative
28. Top of light above bathroom sink	E	35.59, 36.86	Positive	29. Bannister outside laundry	T	ND	ND	Negative	Negative	29. Curtain pole	E	ND	Negative
29. Oxygen saturations probe (outside room)	T	35.99, 35.95	Positive	*Staff office*						30. Wheelbarrow handles	T	ND	Negative
				30. Top of key cabinet	E	37.97, 37.54	37.34	Positive	Suspect	31. Air vent/ extractor (bathroom)	E	37.75	Suspect
										32. Hoist rail (bathroom)	T	ND	Negative
										33. Toilet handle	T	ND	Negative

ND = Not Detected. * Designated common touch point (T) or elevated site (E). † Samples are analysed in duplicate: a positive result means the target gene is detected in both replicates (reported as Cycle threshold (Ct) numbers). If the target gene is identified in only one sample, it is reported as suspect. Ct values reported for N gene target: the higher the Ct value, the lower the concentration of viral RNA. Limit of detection set at Ct 39. NB on the second visit to facility B, some sites were not re-sampled (labelled N/A). Others were combined in one swab (merged cells in the table).

### Settings and population

LTCF sizes ranged in size: there were 40 beds in facility A, which was a self-contained unit within an 80-bedded LTCF, 16 beds in facility B and 13 beds in facility C. The number of occupants was 30 (A), 15 (B) and 12 (C) on the date of onset of the first case. Facility A was a short stay residential unit with clients aged between 55 and 98 years; facility B a residential LTCF for older adults (65 and over) with and without dementia; facility C a residential LTCF for adults with learning difficulties aged between 35 and 88 years. All residents were included in the epidemiological analysis, regardless of age. All residents slept in single occupancy rooms; residents in facilities A and C all had private bathrooms and 13/16 rooms in facility B were en-suite. Characteristics of the three facilities are summarised in Table 2.

**Table 2.** Characteristics of DISinFECT pilot LTCFs and resident populations.

ID	CQC ^ɸ^ rating	No. residents/ no. beds (% occupancy ^*^)	No. floors	Private/ shared bathroom	Care provision	Agency staff	Dependent/ independent	Age range (years)	Walk with purpose ^†^	Prevalence comorbidities ^Ψ^	No. (%) positive sample sites
A	O	30/40 (75.0%)	1	Private	Residential: short stay	Yes - block	Mixed	55- 98	No	23/30 (76.7%)	6/27 (22%)
B	G	15/16 (93.8%)	2	Mixed	Residential: older adults (+/- dementia)	Yes	Mixed	71- 97	Yes	13/15 (86.7%)	17/28 (61%)
C	G	12/13 (92.3%)	2	Private	Residential: adults with Learning Difficulties	No	Mixed	35- 88	Yes	data unavailable	1/31 (3%)

*registered occupancy at time of sampling visit; ^ɸ^CQC = Care Quality Commission, O = Outstanding, G = Good; ^†^one or more resident unable to adhere to self-isolation within private room; ^Ψ^presence of one or more chronic condition (overweight/ obese; chronic respiratory disease; chronic heart disease; dementia; diabetes; hypertension; immunocompromised/ cancer) among registered occupants over the course of the outbreak; Dependent/ independent relates to resident mobility.

### Outbreak trajectories

SARS-CoV-2 attack rates among residents were highest in facility A: 16/30 (53%, of which 15/16 (94%) were symptomatic), followed by facility B: 6/15 (40%, of which 3/6 (50%) symptomatic). Only one resident tested positive in LTCF C (8%): this individual was asymptomatic and had received the first vaccine dose four weeks beforehand. They also had a history of laboratory-confirmed COVID-19 a year previously. A repeat sample taken 10 days after the most recent diagnosis was PCR negative for SARS-CoV-2, nonetheless the individual remained in isolation for 14 days as a precaution. Non-agency staff attack rates were also highest in facility A: 16/60 (27%) followed by facility B: 4/22 (18%) and C: 1/35 (3%). Numbers of agency staff were not available.

**Figure 1.**
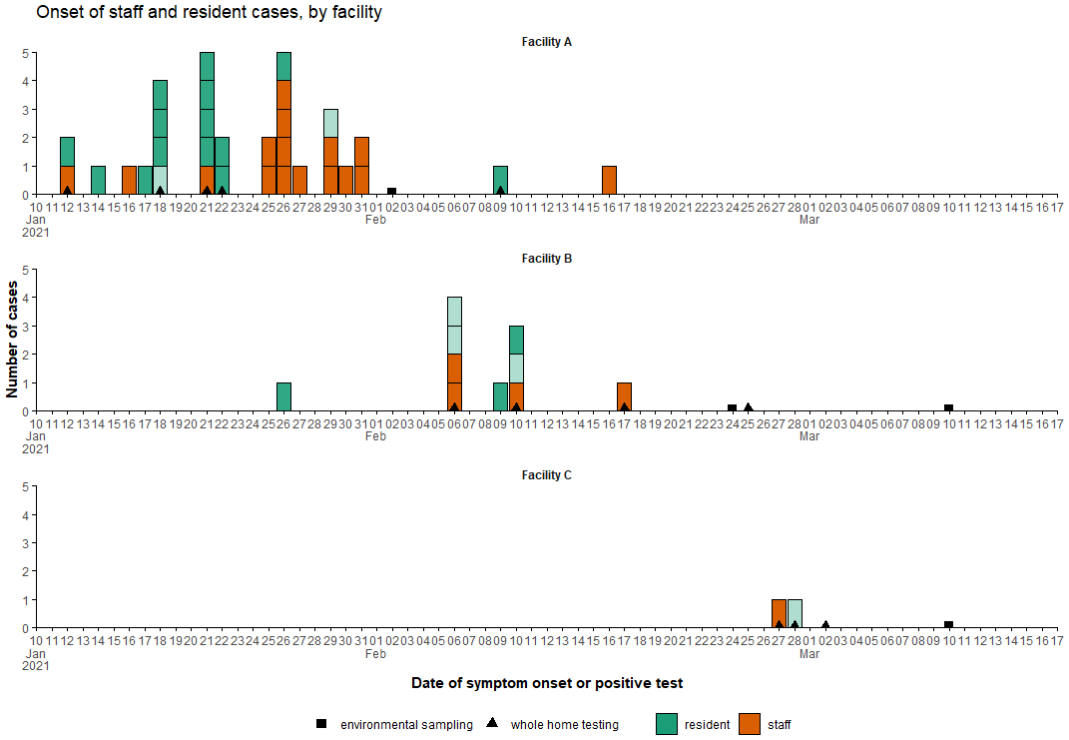
Epidemic curves illustrating case onset in LTCFs A (30 residents), B (15 residents) and C (12 residents). Community case rates peaked at the end of December during the second wave of the UK epidemic.

Duration of outbreak (calculated from the date of first illness onset to 28 days after onset of the final case) was 63, 50 and 30 days for facilities A, B and C respectively. Facilities A and B had residents admitted to hospital (n=5 symptomatic cases and n=3 of which one was symptomatic, respectively). Sadly, there were three COVID-19-related deaths among residents (facility A: n=2, both receiving end-of-life care, one hospitalised; facility B: n=1, hospitalised). Figure 1 illustrates outbreak trajectories.

### Outbreak control measures

Only facility A (the short stay unit) was effectively isolating all residents within their rooms at the time of the sampling visit. Facilities A and C were cohorting staff, allocating them to work exclusively with SARS-CoV-2 positive residents or with SARS-CoV-2 negative residents, and the infected resident in facility C was isolated on a separate floor to the rest of the residents. Staff in all three facilities underwent daily lateral flow (point-of-care) antigen tests for a minimum of five days followed by routine twice weekly testing, as well as weekly PCR testing for SARS-CoV-2 according to national guidance for testing in care homes. All LTCFs were closed to visitors and new admissions for the duration of the outbreak, except for end-of-life visits in facility A. Table 3 summarises outbreak metrics and control measures.

**Table 3.** Outbreak metrics and control measures for DISinFECT pilot LTCFs.

ID	Outbreak metrics					Infection Prevention & Control						COVID-19 vaccine	
	Attack rate residents	Attack rate staff *	Duration ^¥^ (days)	COVID-19 deaths	Hospital admissions	Residents isolating	Staff cohorted	Staff tested ^ф^	Closed to visitors	Enhanced cleaning	PPE ^Ψ^ available	Uptake ^µ^ residents	Uptake ^µ^ staff
A	16/30 (53%)	16/60 (27%)	63	2	5	All	Yes	Yes	Yes ^†^	Yes	Yes	25/30 (83%)	147/183 (80%) ^α^
B	6/15 (40%)	4/22 (18%)	50	1	3	Cases	No	Yes	Yes	Yes	Yes	15/15 (100%)	12/22 (55%)
C	1/12 (8%)	1/35 (3%)	30	0	0	Cases	Yes	Yes	Yes	Yes	Yes	12/12 (100%)	24/25 (96%)

*Approximate staff attack rates, based on numbers of non-agency staff; ^**¥**^Period between onset/ swab dates of index case, and 28 days after illness onset for the final case; ^†^With the exception of end of life visits; ^**ф**^as per PHE guidelines: daily Lateral Flow Tests (LFT) for at least 5 days followed by twice weekly LFT and once weekly PCR testing; ^Ψ^Personal Protective Equipment; ^µ^Receipt of 1 or more dose >=14 days prior to onset in the index case; ^α^Staff vaccination figures provided for whole facility, not just the short stay unit. No dates reported so figures may not accurately represent vaccine-induced immunity

All LTCFs adopted enhanced cleaning protocols in response to the COVID-19 epidemic, with increased frequency and a focus on common touch points. In addition, facility A provided fresh uniforms for staff at the beginning of each shift (laundered on site) and had installed a UV cabinet for treating phones and keys prior to handover. All LTCF managers stated that personal protective equipment (PPE) was available to staff in line with national guidance.

In facilities B and C, 100% of residents had received the first dose of a COVID-19 vaccine more than two weeks prior to outbreak onset. In facility A, 83% of residents had received the first dose of vaccine but just four days before onset of the outbreak. In facility A, 80% of non-agency staff were reported to have received a vaccine, though these figures related to the wider facility and exact vaccination dates were not provided. Facility B reported 55% and facility C 96% of non-agency staff vaccinated with at least one dose more than two weeks prior to the outbreak.

### Observations

Facility A was a modern building with spacious, uncluttered rooms of a uniform layout. Signage was in place to remind staff to clean surfaces and socially distance, and the sampling team observed good adherence to PPE donning and doffing protocols. All residents were isolated in their rooms, and staff wore ‘scrubs’.

Facility B was an older building, once a large house. Residents’ rooms were small and somewhat cluttered with several sampling sites visibly soiled. Several residents were observed using the dining area and lounge (unmasked); staff wore their own clothing.

Facility C was a relatively modern building; rooms were small but uncluttered with fewer soft furnishings than LTCFs A and B. Two residents were observed walking with purpose (unmasked), accompanied by carers; staff wore their own clothing.

Staff wore surgical masks in all three facilities.

### Proportion of sites testing positive and distribution of SARS-CoV-2 RNA

Facility B had the highest proportion of sampling sites testing positive/ suspect for SARS-CoV-2 RNA (PCR positive on one or both duplicates): 17/28 (61%), followed by LTCF A: 6/27 (22%). In facility C, all environmental swabs were negative for SARS-CoV-2 except for one suspect positive from an air extractor in the index case’s bathroom: positivity 1/31 (3%).

A repeat visit to facility B two weeks after the initial sampling visit yielded a much lower proportion of SARS-CoV-2 positive/ suspect sampling sites (4/19; 21%).

The lowest Ct value was 31.71, and only 20% (10/ 51) of values were below Ct 35. Amplification was above the Ct cut-off (39) for one sample (see Table 1).

**Figure 2.**
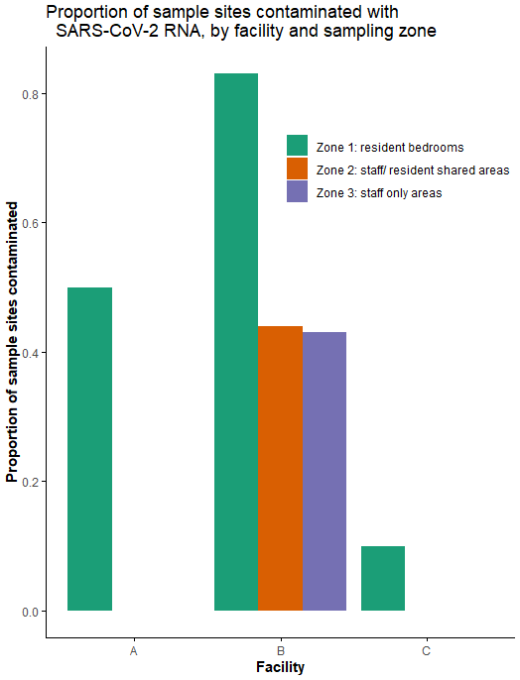
Proportion of sampling sites testing PCR positive for SARS-CoV-2, by sampling zone. There were no zone 2 areas to sample in LTCF A, since all residents were confined to their rooms.

SARS-CoV-2 positive/ suspect surfaces were most common in zone 1 (rooms occupied by residents with active SARS-CoV-2 infection, and equipment used by them), as illustrated in Figure 2.

### Proportion of SARS-CoV-2 positive sites in proximity to a COVID-19 case

**Figure 3.**
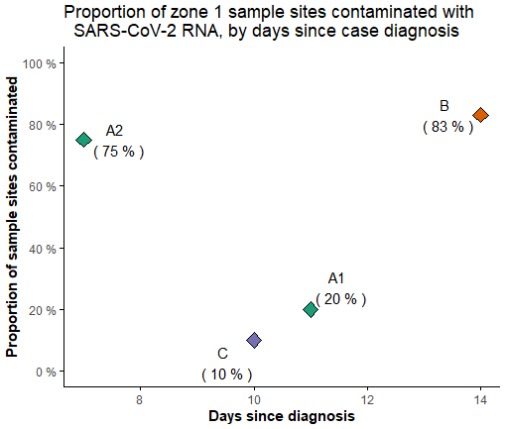
Proportion of sample sites that tested positive for SARS-CoV-2 RNA within the rooms of infected residents. Resident A1 had fever and slight cough and was bed/chair-bound; A2 had a cough and was mobile with a wheelchair; B reported only lethargy, and was independently mobile; C was asymptomatic and independently mobile; this individual’s positive PCR result was unexpected given history of prior infection and vaccination.

Within zone 1, there was significant variation in the proportion of sample sites testing positive/ suspect for SARS-CoV-2 RNA. For example, in facility A two residents’ rooms were sampled: in the first room, 1/5 (20%) of sample sites was ‘suspect positive’ for SARS-CoV-2 RNA and in the second room 6/8 (75%) of sample sites tested positive. Both rooms were similar in size and layout, and subject to the same cleaning protocols. The first room was occupied by an individual who was bed/chair bound, had tested positive for the virus 11 days previously and had fever and a slight cough. The occupant of the second room spent much of their time confined to bed, though was mobile with a wheelchair. This individual tested positive for SARS-CoV-2 infection seven days prior to the visit and had a cough. Figure 3 shows the environmental sample site positivity in relation to the time from onset of illness for the room occupant.

### Proportion of common touch points vs elevated surfaces testing positive for SARS-CoV-2

Overall, the proportion of common touch points testing positive/ suspect for SARS-CoV-2 RNA (15/56; 27%) was slightly lower than the proportion of elevated sites testing positive/ suspect (10/28; 36%), though this was not consistent between LTCFs (see Table 4). Where the proportion of positive common touch points was highest (facility B), a large proportion of elevated sites also tested positive/ suspect for SARS-CoV-2 RNA.

**Table 4.** Proportion of common touch point and elevated sampling sites testing positive for SARS-CoV-2 RNA, by LTCF.

LTCF	No. (proportion) common touch sites positive for SARS-CoV-2	No. (proportion) elevated sites positive for SARS-CoV-2
A	5/17 (29%)	2/10 (20%)
B	10/20 (50%)	6/7 (86%)
C	0/19 (0%)	1/10 (10%)
**Total**	**15/56 (27%)**	**10/28 (36%)**

### Fingertip samples

None of the swabs taken from fingertips of residents with confirmed COVID-19 (facility A: n=2 swabs; facility B: n=3; facility C: n=2) were PCR positive for SARS-CoV-2 RNA.

### WGS of clinical isolates

A small number of outbreak isolates were sequenced (facility A: n=3; facility B: n=3). All were identified as B.1.1.7 (VOC-202012/01), with no E484K substitution detected.

### Staff survey results

Response rates to the electronic staff survey were poor: 11/60 (18%) from facility A, 6/22 (27%) from facility B and 0/35 (0%) from facility C, meaning there was insufficient data to assess clinical and epidemiological risk factors for infection. None of the respondents raised concerns about access to PPE.

## Discussion

The greatest proportions of SARS-CoV-2 positive/ suspect sample sites were found in the immediate vicinity of laboratory-confirmed COVID-19 cases, which is consistent with findings from other studies. ( Onakpoya *et al.*, 2021; Ong *et al.*, 2020; Stevenson *et al.*, 2005) The proportion of SARS-CoV-2 positive/ suspect sites within this sampling zone varied considerably however, even between two rooms with similar layouts and cleaning regimes occupied by individuals with comparable symptom profiles. Levels of environmental contamination around COVID-19 patients evacuated from the Diamond Princess cruise ship and in hospital isolation rooms in Singapore did not correlate with clinical signs of illness either. ( Chia *et al.*, 2020; Santarpia *et al*., 2020) The timing of our samples (7 versus 11 days after symptom onset) may explain some of this variation: Chia *et al.* found more surface contamination in patient isolation rooms during the first week of illness, and associated with lower Ct values from clinical samples, ( Chia *et al.*, 2020) whereas Nelson et al. report no relationship between timing of sampling and proportion of contaminated surfaces in LTCF. ( Nelson *et al.*, 2021) Other studies suggest that there are a myriad of environmental, clinical, behavioural and pathogen factors affecting dispersion of the virus, which must be controlled for in any analysis of infection risk by indirect transmission. ( Moore *et al*., 2021; Onakpoya *et al.*, 2021)

The majority of our positive samples had Ct values >35 which is higher than the average from routine clinical sampling. ( Singanayagam *et al.*, 2020) There is limited value in comparing studies with different protocols, but for context environmental samples from LTCF in British Columbia yielded Ct values of >37.4, ( Nelson *et al.*, 2021) and a systematic review of studies assessing fomitic transmission of SARS-CoV-2 reported surface sample Ct values between 20.8 and 44.1. ( Onakpoya *et al.*, 2021) 

The limit of detection of the Viasure assay is 10 copies/reaction and we consistently detected 5 copies in our standard curve. Since we saw no amplification in our negative extract samples or qPCR no-template controls we are confident the late amplification we detected in COVID-19 outbreak environmental samples is genuine detection of SARS-CoV-2 RNA

The fact that fingertip swabs were all PCR negative for SARS-CoV-2, despite observing infected residents touching contaminated areas, was surprising: people typically touch their nose, eyes and mouth more than 20 times per hour and an experimental study suggested that the virus can persist on skin for at least 8 hours at body temperature. ( Harbourt *et al*., 2020; Kwok *et al*., 2015) Sampling may have occurred too late to detect viral shedding, since participants were between seven and 14 days of diagnosis, and the dispersal of virus on surfaces will not have been uniform. It is also possible that residents applied hand sanitiser unobserved, that the sampler did not apply sufficient pressure or friction to pick up viral RNA, or that flocked swabs have a very low recovery efficiency on skin. ( Mbithi *et al*., 1992)

Notably, facility A experienced the highest attack rates despite implementing more comprehensive IPC and cleaning measures compared to facilities B and C. Residents in facility A were more susceptible to infection than those in facilities B and C, having only received the first dose of vaccine shortly before the onset of the outbreak. ( Shrotri *et al*., 2021; Tenforde *et al*., 2021) Facility A also had a higher rate of admissions from the local hospitals and these factors, as well as the relatively large size of the facility, may have increased the probability of multiple introductions of the virus to the premises. ( Burton *et al*., 2020; Shallcross *et al*., 2021) Poor response rates to the staff survey, reflecting extreme work pressures, meant that analysis of individual-level infection risk factors was not possible.

High attack rates may also have reflected community case rates, which peaked at the end of December and remained high through January (see Figure 1 for pilot LTCF outbreak trajectories). This surge in case rates was fuelled by emergence of the more transmissible B.1.1.7 Alpha variant, which quickly entered English LTCFs. ( Krutikov *et al*., 2021) At this time a substitution at the E484K location of the receptor binding domain also appeared, raising concerns that the virus might evade the host immune response. ( Wise, 2021) Six clinical samples were sequenced and were all of the B.1.1.7 Alpha variant with no E484K substitution, however we cannot exclude the possibility that multiple strains of the virus contributed to these outbreaks.

Results from surface swabbing provided some reassurance that facility A (with staff cohorting and enhanced IPC measures) was effectively containing the environmental spread of the virus, in contrast to LTCF B (without cohorting) in which viral RNA was widely distributed. Repeat sampling 14 days after the initial visit to facility B yielded a much lower proportion of SARS-CoV-2 positive/suspect sites. Since the first visit corresponded with the end of the final case’s infectious period and no further cases of COVID-19 were identified it seems unlikely that anyone in the facility was actively shedding virus at the second sampling visit. Our observations could reflect the effectiveness of cleaning protocols introduced between sampling visits or of swabbing at the first round of sampling, and/ or degradation of viral RNA over a 14 day period. ( Onakpoya *et al*., 2021; van Doremalen *et al*., 2020) Facility C was the only one to isolate its resident case on a separate floor/wing which may have helped reduce egress to other areas within the LTCF. However, this individual’s history of vaccination and prior infection, and a negative repeat PCR test suggest that the diagnosis was a false positive and they were not shedding SARS-CoV-2 at the time of sampling.

Elevated sampling sites, being cleaned less regularly, may be a more pragmatic means of SARS-CoV-2 detection than common touch points: of the four sites that remained positive/suspect for SARS-CoV-2 on a repeat visit to facility B, three were elevated. Reactive sampling, as applied in this pilot, will not distinguish between historic and current viral shedding but there is evidence that levels of surface contamination with SARS-CoV-2 RNA mirror contemporaneous levels of airborne SARS-CoV-2 RNA. ( Cherrie *et al*., 2021; Dumont-Leblond *et al*., 2021; Zhou *et al.*, 2021) Air vents may be useful sentinel sampling points since three of four air vents in facility B tested positive for SARS-CoV-2 RNA, and in facility C the air vent was the only sampling site that tested suspect positive. Similar observations are reported from sampling ventilation grates in the Diamond Princess COVID-19 quarantine rooms and in COVID-19 isolation rooms in Nebraska, and respiratory viruses have been isolated from air filters in aeroplanes and large public buildings. ( Goyal *et al*., 2011; Korves *et al*., 2011; Santarpia *et al*., 2020) It is interesting that none of the air vents sampled from facility A tested positive for SARS-CoV-2 RNA, including one in a room that was otherwise quite heavily contaminated. This could have been an artefact of different sampling techniques, or reflect the design of the air vents, which were circular with a single ring opening rather than a slatted grate, though the vent that tested suspect positive in facility C was of the same circular design. Facility A may also have been better ventilated than the other facilities, which has been associated with lower levels of surface contamination, ( Dumont-Leblond *et al.*, 2021) however this seems unlikely given positive results in other elevated sites in the building.

The uncluttered environment in facility A, in reducing build-up of dust, may have helped limit environmental spread of SARS-CoV-2: there is some evidence that respiratory droplets containing SARS-CoV-2 are adsorbed to dust and particulate matter. ( Andree, 2020; Conticini *et al*., 2020; Qu *et al*., 2020; Renninger *et al*., 2021; Setti *et al*., 2020; Travaglio *et al*., 2021). The presence of dust can also indicate poor ventilation, which increases the likelihood of aerosol deposition; ( Dumont-Leblond *et al.*, 2021) – this merits further investigation.

### Limitations

This pilot has limitations, including small sample size, lack of control sites and heterogeneity of sample sites. Our interpretation of results is speculative and intended to generate hypotheses rather than answer questions.

Between-site variation in layout and infrastructure means that sampling frames cannot be entirely standardised. In addition, there may be a tendency to oversample areas that are visibly soiled; a weak correlation was found between the number of samples taken from COVID-19 isolation rooms in Nebraska and the proportion of samples that tested positive for SARS-CoV-2 RNA. ( Santarpia *et al.*, 2020). Technique may also vary between samplers, though only two individuals conducted sampling in this study which should have minimised discrepancies. We did not capture timings for cleaning activity which could explain some of the variation we saw in environmental contamination, though variation was also observed between elevated sites which were unlikely to have been cleaned as part of daily routine. Results from surface swabs represent a snapshot in time and cover a small fraction of the LTCF environment so we may not have accurately captured overall levels of environmental contamination. Among other things these may have been influenced by trends in community prevalence of COVID-19 (affecting risk of importation), expansion of new variants, and the effects of vaccination (impacting viral shedding and transmissibility).

We chose to use flocked swabs and sponges based on a laboratory-based comparison of recovery efficiencies from surfaces inoculated with low concentrations of SARS-CoV-2 RNA (Evaluation of SARS-CoV-2 environmental surface sampling protocols conducted by UKHSA for the PROTECT COVID-19 National Core Study on transmission and environment). We did not assess recovery efficiency in the field so do not know how sensitive our sampling methods were when applied in LTCF. More work is needed to validate limits of detection and assess sensitivity of environmental sampling for SARS-CoV-2 in LTCF.

We did not include field controls in our sampling strategy and cannot exclude the possibility that there was contamination or cross-contamination of samples.

We were unable to confirm whether the diagnostic test for the single resident case in facility C was a true positive, therefore the SARS-CoV-2 RNA detected in this individual’s rooms may have been residual from previous occupants or their carers. This bias also applies to the other facilities to some extent since all were likely to have been exposed to the virus at some point prior to the outbreak.

Sequencing data were only available for a minority of outbreak samples therefore we were unable to assess whether new or multiple strains contributed to these outbreaks.

## Conclusions and recommendations

We have successfully recruited three pilot LTCFs to the DISinFECT pilot and recovered SARS-CoV-2 RNA from a high proportion of surfaces around individuals with a laboratory-confirmed infection. Elevated surfaces had relatively high positivity so sampling these areas may be a simple way to detect pathogens that have been airborne, though we cannot infer infection risk from environmental results. Our experiences highlight that LTCF staff can be overburdened with information requests during outbreaks. There is therefore a need for efficient and parsimonious data collection tools to support the interpretation of environmental surveillance data.

In summary: there is a need for tools to help detect outbreaks in LTCF and to optimise IPC in these settings. This pilot suggests that further work is justified to assess the feasibility and utility of environmental sampling for infectious disease surveillance in LTCF.

## Data Availability

To preserve anonymity of LTCF residents and staff, the study data are stored on a secure drive hosted by the UK Health Security Agency (UKHSA) Field Services South West. Access to personal identifiable data is restricted to personnel responsible for outbreak investigations. PCR results from environmental sampling are available via the Open Science Framework via the following link: https://doi.org/10.17605/OSF.IO/DE2ZF. Open Science Framework: COVID-19: Detecting Indirect Spread in Facilities for Enhanced Care sTudy (COVID-19: DISinFECT). Investigating environmental epidemiology of SARS-CoV-2 in long term care facilities in England. Protocol v4.1. https://doi.org/10.17605/OSF.IO/3QN9Z ( Kwiatkowska & Ready, 2021) This project contains the following files: - DISinFECT_Protocol_OSF.pdf. The protocol for this research study. - DISinFECT_staffsurvey.pdf. The electronic survey distributed to staff to collect information on epidemiological and clinical risk factors for SARS-CoV-2 infection. - DISinFECT_Tools_line list_v6.xlsx. The data collection template for LTCF residents: clinical and epidemiological risk factors for SARS-CoV-2 infection. - DISinFECT_Tools_setting log_v2.xlsx. The data collection template for details of LTCF layout and staffing arrangements. Data are available under the terms of the Creative Commons Attribution 4.0 International license (CC-BY 4.0).
