# Supplementary material for: Environmental sampling for SARS-CoV-2 in long term care facilities: lessons from a pilot study
Source: Wellcome Open Res. 2023 Jun 28;6:235. Originally published 2021 Sep 15. [Version 2] doi: 10.12688/wellcomeopenres.17047.2 (PMC10891430; doi:10.12688/wellcomeopenres.17047.2)
Supplement: Peer review history

# Reviewer report: Reviewer response for version 2

**DOI:** 10.21956/wellcomeopenres.21727.r71678

**Published:** 2024-02-20

**Authors:** Hiromu Yoshida

**Affiliations:** 1 National Institute of Infectious Diseases, Musashimurayama-shi, Tokyo, Japan

The authors report on SARS-CoV-2 surface contamination status during the COVID-19 outbreak in three LTCFs. Environmental surveillance by wiping surfaces in the facility could be a potential tool for monitoring infectious diseases as a non-invasive method. It could support testing by specimen collection from residents and staff within the LTCF. As SARS-CoV-2 is a respiratory infection, information on which environmental surfaces in the facility are contaminated with virus in droplets would be useful for infection control.

 The results of the survey showed that the viral genome could be detected in less touched, elevated areas as well as in areas touched by people, suggesting that these less touched areas are potential candidates as sites for identifying the presence of an infected person, unaffected by daily surface cleaning in the LTCF.

 At the same time, the results also indicated that aerosol control from infected individuals would be important. However, as they pointed out in the limitations section, even if the wipe tests were positive, it could not imply infectivity. In addition, because Ct value in most positive cases was high, the results would be interpreted with various speculations, e.g. it might have simply been tracking past contamination residues.

 In other words, it is difficult to confirm indirect transmission from these data.

 However, the presence of viral genome on various surfaces in the LTCF reaffirms the need for infection control measures corresponding to aerosol exposure and close contact infection.

 As the authors mentioned, thus survey would increase workload for staff in LTCF during the outbreak. If possible, it would be valuable to indicate how the survey results had influenced infection control measures in the facility.

 Below minor points could be clarified by the authors:

 Sampling:

 Although the authors mentioned two staffs were in charge for sampling, this information would move to "sampling" in M&M section.  The authors should mention for measures against cross-contamination for PCR test in sampling.

 Outbreak control measures:

 In this study, most PCR tests indicate high number of Ct value. How is the interval between sampling and surface cleaning at facilities A, B, and C?  If records not available, please discuss whether” enhanced cleaning” would relate to detect viral genome by PCR test or not in the limitation section.

 Result:

 After the results of the environmental sampling were returned to each LTCF, what kinds of action was taken by LTCF? If possible, could you describe it.

 Table 1:

 LTCF B. Line7. 2nd visit. “Negative” is correct?

 Please check again whether numbers of samples in Table are same as those in result section.

 Figure 1:

 The result for environmental sampling could be helpful to understand a prevalence of contaminated surface at LTCF. Could the authors indicate positive rate in each sampling date in figure?

Is the work clearly and accurately presented and does it cite the current literature?

Yes

If applicable, is the statistical analysis and its interpretation appropriate?

Not applicable

Are all the source data underlying the results available to ensure full reproducibility?

Yes

Is the study design appropriate and is the work technically sound?

Yes

Are the conclusions drawn adequately supported by the results?

Yes

Are sufficient details of methods and analysis provided to allow replication by others?

Yes

Reviewer Expertise:

environmental surveillance

I confirm that I have read this submission and believe that I have an appropriate level of expertise to confirm that it is of an acceptable scientific standard.

# Reviewer report: Reviewer response for version 2

**DOI:** 10.21956/wellcomeopenres.21727.r61519

**Published:** 2023-07-26

**Authors:** Timothy R. Julian

**Affiliations:** 1 Eawag, Swiss Federal Institute of Aquatic Science and Technology, Dübendorf, Switzerland

The authors have thoughtfully and rigorously updated the methods to clarify processes and results, and modified discussions to align interpretation with the data. The result is a manuscript that reaffirms that environmental sampling of surfaces can be used to detect pathogens, including SARS-CoV-2. Further, environmental sampling in long term care facilities can offer a non-invasive surveillance approach for the emergence or spread of a disease in the facility. The results is a comprehensive case study offering both methodologies and insights into fomite-mediated disease surveillance.

 Notably, the authors also investigated a variety of surface types, including sites elevated (>1.5m) off the ground and note more frequent detection at these sites. This finding offers additional insights into the potential aerosolized spread of SARS-CoV-2 prior to deposition, and suggests non-traditional sampling of surfaces beyond those frequently contacted may be beneficial when surface testing is focused on surveillance, as opposed to assessing potential for transmission risks.

 The authors highlight the need for continued work on this subject, and point to specific areas where advances would be warranted. Quantification of pathogens from surfaces is once such area they note, as quantity drives transmission risks, though may be less important to accurately determine for surveillance purposes where presence/absence measured here may be sufficiently informative.

Is the work clearly and accurately presented and does it cite the current literature?

Yes

If applicable, is the statistical analysis and its interpretation appropriate?

Not applicable

Are all the source data underlying the results available to ensure full reproducibility?

No

Is the study design appropriate and is the work technically sound?

Partly

Are the conclusions drawn adequately supported by the results?

Partly

Are sufficient details of methods and analysis provided to allow replication by others?

Partly

Reviewer Expertise:

Environmental Microbiology, Environmental Virology, Exposure Assessment, Risk Assessment

I confirm that I have read this submission and believe that I have an appropriate level of expertise to confirm that it is of an acceptable scientific standard.

# Reviewer report: Reviewer response for version 1

**DOI:** 10.21956/wellcomeopenres.18828.r45883

**Published:** 2021-10-15

**Authors:** Timothy R. Julian

**Affiliations:** 1 Eawag, Swiss Federal Institute of Aquatic Science and Technology, Dübendorf, Switzerland

**Overview:**

 The study describes epidemiology of SARS-CoV-2 outbreaks in three long term care facilities (LTCF) during SARS-CoV-2 outbreaks in the South West of England from January to March 2021, with a focus on the results of surface sampling. The topic is importantly and timely, highlighting the potential use of environmental sampling to both inform disease prevalence (surveillance) and to identify risks of transmission (fomite-mediated transmission).

 The study identifies extensive contamination of SARS-CoV-2 RNA in one of the three sites, and almost no detectable contamination at one of the sites. Further, the study concluded that elevated surfaces – or surfaces with sufficient height they would not be expected to be cleaned regularly – were more likely to have detectable RNA than other sites. The study is well-written, describing a case study of contamination in LTCFs which are an important site of concern for transmission of SARS-CoV-2. Further, the study is embedded within a larger, more extensive study which may provide important insights on transmission routes of SARS-CoV-2 in these settings.

 The manuscript should minimally be updated to include more details on methodology of surface sampling, as the text description is limited and hinders interpretation of the results. Additionally, laboratory controls (field blank, extraction blank, no template controls, positive controls, inhibition testing, recovery efficiency experiments) are not described, making it unclear the extent to which results may be due to sample contamination (or cross-contamination) as well as efficacy of the protocol. Given this is environmental sampling typically characterized by low concentrations and low positivity rates (as reflected in the results), adequate controls are particularly important. 

 Additional improvements (or discussion at least) of a lack of a standard curve is warranted, given that it hinders experimental interpretation, particularly discussions of concentrations of SARS-CoV-2 on surfaces and the associated risks of contacts. Synthetic SARS-CoV-2 RNA is commercially available, and would provide a sufficient standard for quantification of samples.

 An additional update is that authors should consider modifying the manuscript discussion and conclusions to more closely align with the results. A specific example is that the study investigated three LTCFs and attempts to draw conclusions on results as a function of cleaning regime: such inference would require a larger sample size with multiple facilities with variable cleaning and quarantining regimes to rule out location-specific effects not associated with cleaning.

**Detailed Comments:**

**Abstract:**

- “potential to assess infection risk from SARS-CoV-2 positive surfaces” - should be connected to methods for this approach, unclear how this would be done.
- Define “elevated” here and in Methods.
- “could be associated with implementation” – this is speculation, and was not specifically tested, so suggest authors remove this from the abstract. 

**Introduction:**

- “touch-points and elevated surfaces” – how did authors conclude they should test elevated surfaces? Is prior literature available here that could be referenced?
- Author discussion of COVID-19: DISinFECT suggests a project larger than the study results presented here, but it is unclear. Author clarification on the project DISinFECT and its relationships to this study would be helpful and interesting. 

**Methods:**

- “complete a consent form” – suggest “written consent form”.
- Substantially more information is recommended on collecting samples. Detection/Non-detection of viral RNA on surfaces is influenced by the sensitivity of the methods, which includes sample collection, RNA extraction, and RNA detection. Authors should consider reporting material used (not only swabs, but also eluent volume and material, volume of material subsequently processed, RNA extraction methods, including volumes eluted (if applicable), and full details of the QPCR methodology. Inhibition may also influence sample detection, and some indication of qPCR inhibition testing should be included. Indeed, less frequently contacted surfaces (like elevated surfaces) may have more dust and thus may be more inhibited? 
- Bustin *et al.* 2009 MIQE Guidelines ^1^ provide a motivation and framework for reporting on data relying on qPCR. Additionally, authors should report all controls used, including positive controls (source and concentration), field blanks, extraction blanks, and no template controls. Authors should also conduct, or report on previously studies that conducted, SARS-CoV-2 or surrogate virus RNA from surfaces to provide an estimate of recovery efficiency. Environmental sampling relying on detection of low concentrations should demonstrate sufficient use of controls to rule out contamination and cross contamination of samples, and recovery efficiency to provide insights into relative magnitude of surface contamination.
- How was a Ct value of 39 chosen as a cut-off? How does this value compare to no template controls? See MIQE guidelines ^1^ for guidance on Limits of Detection. Authors could consider motivating why no standard curve was included to estimate concentrations.
- On mention of WGS, it was unclear in the methods that this meant clinical isolates and not environmental samples. Suggest authors clarify. Also, authors should specify directly, for example through referencing, what protocols were followed, what primers sets were used, for sequencing. 
- Figure 1 provides minimal novel insights into the study, authors could consider replacing with more descriptive figure covering all specific locations tested, with further information on their classification (elevated or not).

**Results**

- Term “home” is used and could be replaced with LCTF.
- Table 1 – in one negative sample, a threshold Ct value is reported. Similar, positive Ct is reported for all samples. Consider reporting Ct for all samples as a separate column.
- Table 2 – a helpful table for comparing locations, is it possible to add the percent of surfaces that were positive at each site?
- Figure 2- why is C not included in this trajectory?
- “Concentrations of SARS-CoV-2 RNA” – in the absence of standard curves and recovery efficiency experiments, there is an obscure relationship between Ct values and true (unobserved) concentrations on surfaces. Suggest authors avoid discussing or inferring concentrations and instead focus on detection/non-detection.
- “lower than the proportion of sites” – should this say elevated sites?
- Fingertip samples – repeat how many samples were tested here.
- Figure 3 – suggest updating legend descriptions from Zone 1-3 to more detailed descriptions provided in legend to make it easier to read.
- Figure 4 – unclear why there are two y-axes here since there are only four data points. Suggest authors place days since illness as a number above the bar charts. An alternative, if the point is to visualize the relationship (or lack of) between days since illness and positivity rate, is to plot a scatter plot with days since illness on x-axis and positivity rate on y-axis and label each point with A1, A2, B, or C with text labels.

**Discussion**

- “with findings from other studies” – authors should reference these studies in this sentence, only one study is currently referenced.
- “can detect the presence of an infected individual” – although conceptually the authors demonstrate detection in rooms with infected patients, the study does not evaluate the specificity or sensitivity of the approach: how often in an infected patients room or other building area is SARS-CoV-2 detected in the room? How often is SARS-CoV-2 detected in uninfected patients rooms or other building areas? What are the false negative/false positive rates? These values are necessary to determine the feasibility of this approach in environmental surveillance.
- “Consequently, there was insufficient epidemiological… “ – I am a bit skeptical that survey results could provide insight into the role of fomites vs. long range aerosol vs. close contact in disease transmission. Can the authors further describe how this could be done?
- “The fact that fingertip swabs…”, could also be very low methodological recovery. This should be tested or at least discussed in the context of other work on recovery efficiency. An alternative is spatial heterogeneity (in addition to temporal heterogeneity discussed by authors).
- “may have been residual” – or analytical false negatives.
- “This pilot demonstrates…” - the efficacy of surface swabbing to determine efficacy of IPC measures should rely on substantially more than three LCTFs, given that the variation in site-specific contamination may be caused by factors other than IPC. The sample size is too small to clearly demonstrate this. Suggest authors rephrase this point to highlight that future studies could use these methodology to explore impacts of IPC on surface contamination.

**Comments on specific elements of the review:**

**Technically Sound / Methods and Analysis:**

- As discussed, more data on methods and associated results of controls would be beneficial.

**Statistical Analysis:**

- The analysis presented here is primarily qualitative given the pilot nature, small sample size, and heterogeneity in results.

**Data Availability:**

- The authors present much of their data in the manuscript, and offer the rest (non-identifiable) on contact. Although this is generally sound, suggest authors make non-identifiable data freely available, in particular a modified version of Table 1 that includes all Ct values (not the mean, SD).

Is the work clearly and accurately presented and does it cite the current literature?

Yes

If applicable, is the statistical analysis and its interpretation appropriate?

Not applicable

Are all the source data underlying the results available to ensure full reproducibility?

No

Is the study design appropriate and is the work technically sound?

Partly

Are the conclusions drawn adequately supported by the results?

Partly

Are sufficient details of methods and analysis provided to allow replication by others?

Partly

Reviewer Expertise:

Environmental Microbiology, Environmental Virology, Exposure Assessment, Risk Assessment

I confirm that I have read this submission and believe that I have an appropriate level of expertise to state that I do not consider it to be of an acceptable scientific standard, for reasons outlined above.

## References

[1] : The MIQE guidelines: minimum information for publication of quantitative real-time PCR experiments. *Clin Chem* .2009;55(4) : 10.1373/clinchem.2008.112797 611-22 10.1373/clinchem.2008.112797 19246619

# Response

**Published:** 2023-06-27

**Authors:** Rachel Kwiatkowska

**Affiliations:** University of Bristol, UK

Thank you for the thorough and constructive review, please see our responses below. Hopefully, the amended manuscript will clarify our methods. Considering the limitations of the study, we have modified the discussion and conclusions to flag points of interest/ further investigation without drawing too much inference from this small number of observations.

**Abstract**

 1. “potential to assess infection risk from SARS-CoV-2 positive surfaces” - should be connected to methods for this approach, unclear how this would be done.

- Thanks for pointing this out. We’ve updated the wording to: ‘ *Collection of clinical and epidemiological data to provide context for environmental findings*.’

2. Define “elevated” here and in Methods.

- Defined as a surface >1.5m above ground, see amended abstract and methods > sampling > approach.

3. “could be associated with implementation” – this is speculation, and was not specifically tested, so suggest authors remove this from the abstract. 

- Agreed – we have removed this statement.

**Introduction:** 4. “touch-points and elevated surfaces” – how did authors conclude they should test elevated surfaces? Is prior literature available here that could be referenced?

- This decision was based on our own observations from sampling in healthcare settings, and findings from other studies. We’ve added this information to paragraph 2 of the introduction.

5. Author discussion of COVID-19: DISinFECT suggests a project larger than the study results presented here, but it is unclear. Author clarification on the project DISinFECT and its relationships to this study would be helpful and interesting. 

- Thanks, we’ve added additional detail to the final paragraph of the introduction.

**Methods:** 6. “complete a consent form” – suggest “written consent form”

- Amended.

7. Substantially more information is recommended on collecting samples. Detection/Non-detection of viral RNA on surfaces is influenced by the sensitivity of the methods, which includes sample collection, RNA extraction, and RNA detection. Authors should consider reporting material used .. .. swabs,

- Please see ‘sampling’ section, paragraph 2...

eluent volume and material, volume of material subsequently processed, RNA extraction methods, including volumes eluted (if applicable)

- Added to the beginning of ‘laboratory analysis’ section.

..full details of the QPCR methodology.

- Added to ‘laboratory methods’, second paragraph. We did not perform qPCR on sponge samples, and have added this to our limitations.

8. Inhibition may also influence sample detection, and some indication of qPCR inhibition testing should be included. Indeed, less frequently contacted surfaces (like elevated surfaces) may have more dust and thus may be more inhibited? 

- An important point, thank you. See details of inhibition testing in ‘laboratory methods’, paragraph 4. We detected no evidence of inhibition. Interesting point regarding dust as an inhibitor: colleagues have processed quite a lot of samples from heavily soiled areas in public transport, and still detected viral RNA.
- Please see ‘laboratory methods’ section for details of positive controls, extraction blanks and no template controls. Field controls/ blanks were not used - we have listed this among the study limitations.

9. Authors should also conduct, or report on previously studies that conducted, SARS-CoV-2 or surrogate virus RNA from surfaces to provide an estimate of recovery efficiency.

- See discussion - we have referenced (unpublished) UKHSA data on recovery efficiency of flocked swabs and sponges, which informed our choice of sampling materials. We did not attempt to calculate recovery efficiency in the field and have added this to the study limitations.

10. Environmental sampling relying on detection of low concentrations should demonstrate sufficient use of controls to rule out contamination and cross contamination of samples, and recovery efficiency to provide insights into relative magnitude of surface contamination

- Thank you for the detailed explanation and reference to MIQE guidelines. We hope that the additional methodological detail and inclusion of caveats improves the quality of the paper.

11. How was a Ct value of 39 chosen as a cut-off? How does this value compare to no template controls? See MIQE guidelines ^1^ for guidance on Limits of Detection. Authors could consider motivating why no standard curve was included to estimate concentrations.

- We followed the example of other environmental sampling studies in healthcare and LTCF when defining the cut-off at Ct 39 – we’ve referenced these in methods > laboratory analysis > analysis

12. On mention of WGS, it was unclear in the methods that this meant clinical isolates and not environmental samples. Suggest authors clarify. Also, authors should specify directly, for example through referencing, what protocols were followed, what primers sets were used, for sequencing. 

- Thanks, we’ve clarified that these were clinical isolates. Sequencing was carried out externally, through a consortium of laboratories operating in the COVID-19 response; unfortunately we do not have access to the protocols used.

13. Figure 1 provides minimal novel insights into the study, authors could consider replacing with more descriptive figure covering all specific locations tested, with further information on their classification (elevated or not).

- We have decided to leave this figure out, having attempted a more comprehensive visual which got quite messy.

**Results** 14. Term “home” is used and could be replaced with LCTF.

- All references to (care) home corrected to LTCF.

15. Table 1 – in one negative sample, a threshold Ct value is reported. Similar, positive Ct is reported for all samples. Consider reporting Ct for all samples as a separate column.

- N gene Ct values added for all samples. 

16. Table 2 – a helpful table for comparing locations, is it possible to add the percent of surfaces that were positive at each site?

- Good idea, added.

17. Figure 2- why is C not included in this trajectory?

- We have now added the two data points for facility C.

18. “Concentrations of SARS-CoV-2 RNA” – in the absence of standard curves and recovery efficiency experiments, there is an obscure relationship between Ct values and true (unobserved) concentrations on surfaces. Suggest authors avoid discussing or inferring concentrations and instead focus on detection/non-detection.

- Noted. We have simply commented on high Ct values without inference re: viral/ RNA concentrations. 

19. “lower than the proportion of sites” – should this say elevated sites?

- Yes. Corrected.

20. Fingertip samples – repeat how many samples were tested here.

- Amended.

21. Figure 3 – suggest updating legend descriptions from Zone 1-3 to more detailed descriptions provided in legend to make it easier to read.

- Legend updated.

22. Figure 4 – unclear why there are two y-axes here since there are only four data points. Suggest authors place days since illness as a number above the bar charts. An alternative, if the point is to visualize the relationship (or lack of) between days since illness and positivity rate, is to plot a scatter plot with days since illness on x-axis and positivity rate on y-axis and label each point with A1, A2, B, or C with text labels.

- Good suggestion – we’ve reproduced this as a scatter plot.

**Discussion** 23. “with findings from other studies” – authors should reference these studies in this sentence, only one study is currently referenced.

- Additional references added.

24. “can detect the presence of an infected individual” – although conceptually the authors demonstrate detection in rooms with infected patients, the study does not evaluate the specificity or sensitivity of the approach: how often in an infected patients room or other building area is SARS-CoV-2 detected in the room? How often is SARS-CoV-2 detected in uninfected patients rooms or other building areas? What are the false negative/false positive rates? These values are necessary to determine the feasibility of this approach in environmental surveillance.

- You’re right, more work needed – we’ve removed this sentence and added to the limitations: ‘ *More work is needed to validate limits of detection and assess sensitivity and specificity of environmental sampling for SARS-CoV-2 in LTCF’*

25. “Consequently, there was insufficient epidemiological… “ – I am a bit skeptical that survey results could provide insight into the role of fomites vs. long range aerosol vs. close contact in disease transmission. Can the authors further describe how this could be done?

- We have deleted this sentence since we did not intend to conduct statistical analyses as part of the DISinFECT study.

26. “The fact that fingertip swabs…”, could also be very low methodological recovery. This should be tested or at least discussed in the context of other work on recovery efficiency. An alternative is spatial heterogeneity (in addition to temporal heterogeneity discussed by authors).

- Thanks, yes. We mention recovery efficiency here and discuss in the limitations. Re: spatial heterogeneity, the next sentence now reads: *‘Sampling may have occurred too late to detect viral shedding, since participants were between seven and 14 days of diagnosis, and the distribution of virus on surfaces will not have been uniform’*

27. “may have been residual” – or analytical false negatives.

- Do you mean a false positive environmental swab, or false negative from the room’s occupant? We’ve implied the latter.

28. “This pilot demonstrates…” - the efficacy of surface swabbing to determine efficacy of IPC measures should rely on substantially more than three LCTFs, given that the variation in site-specific contamination may be caused by factors other than IPC. The sample size is too small to clearly demonstrate this. Suggest authors rephrase this point to highlight that future studies could use these methodology to explore impacts of IPC on surface contamination.

- Thanks, we’ve modified this paragraph as suggested.
